# *Psoralea corylifolia* L. Ameliorates Collagen-Induced Arthritis by Reducing Proinflammatory Cytokines and Upregulating Myeloid-Derived Suppressor Cells

**DOI:** 10.3390/life11060587

**Published:** 2021-06-21

**Authors:** Fu-Tzu Pai, Cheng-You Lu, Chia-Hsin Lin, John Wang, Ming-Cheng Huang, Chuan-Teng Liu, Ying-Chyi Song, Cheng-Lung Ku, Hung-Rong Yen

**Affiliations:** 1 Graduate Institute of Clinical Medical Sciences, College of Medicine, Chang Gung University, Taoyuan 33302, Taiwan; D0100501@cgu.edu.tw; 2 Research Center for Traditional Chinese Medicine, China Medical University Hospital, Taichung 40447, Taiwan; u101030078@cmu.edu.tw (C.-H.L.); t83320@mail.cmuh.org.tw (C.-T.L.); song@mail.cmu.edu.tw (Y.-C.S.); 3 Cardiovascular and Mitochondrial Related Disease Research Center, Hualien Tzu Chi Hospital, Buddhist Tzu Chi Medical Foundation, Hualien 97002, Taiwan; cylu@tzuchi.com.tw; 4 School of Chinese Medicine, College of Chinese Medicine, China Medical University, Taichung 40402, Taiwan; D19957@mail.cmuh.org.tw; 5 Department of Pathology, China Medical University Hospital, Taichung 40447, Taiwan; D96085@mail.cmuh.org.tw; 6 Department of Chinese Medicine, China Medical University Hospital, Taichung 40447, Taiwan; 7 Graduate Institute of Integrated Medicine, College of Chinese Medicine, China Medical University, Taichung 40402, Taiwan; 8 Chinese Medicine Research Center, China Medical University, Taichung 40402, Taiwan; 9 Department of Biotechnology, Asia University, Taichung 41354, Taiwan

**Keywords:** *Psoralea corylifolia* L., collagen-induced arthritis, myeloid-derived suppressor cells, proinflammatory cytokines, rheumatoid arthritis

## Abstract

*Background*: Rheumatoid arthritis is an autoimmune disease that may lead to severe complications. The fruit of *Psoralea corylifolia* L. (PCL) is widely used in traditional Chinese medicine as a well-known herbal treatment for orthopedic diseases. However, there is a lack of studies of its effects on rheumatoid arthritis. The purpose of the study was to investigate the effects and mechanisms of concentrated herbal granules of PCL on rheumatoid arthritis to provide some insights for future development of new drug for the treatment of rheumatoid arthritis. *Methods*: We used collagen-induced arthritis (CIA) DBA/1J mice as an experimental model to mimic human rheumatoid arthritis. The mice were immunized with collagen on days 0 and 21 and then orally administered 200 mg/kg/day PCL on days 22–49. Starch was used as a control. The mice were sacrificed on day 50. Clinical phenotypes, joint histopathology, and immunological profiles were measured. *Results*: Compared to the CIA or CIA + Starch group, the CIA + PCL group had significantly ameliorated clinical severity and decreased paw swelling. Histopathological analysis of the hind paws showed that PCL mitigated the erosion of cartilage and the proliferation of synovial tissues. There were significant differences in the levels of TNF-α, IL-6 and IL-17A, as measured by ELISA, and the percentages of CD4 + IL-17A+, CD4 + TNF-α+, CD4 + IFN-γ+ T cells. Furthermore, we also found that in mice treated with CIA + PCL, the percentage and number of bone marrow-derived suppressor cells (MDSCs; Gr1+ CD11b+) increased significantly. *Conclusions*: We provided evidence for the potential antiarthritic effects of PCL through the inhibition of inflammation and increase of MDSCs. These findings indicate that PCL may be a promising therapeutic herb for the treatment of rheumatoid arthritis.

## 1. Introduction

Rheumatoid arthritis (RA) is a common systemic autoimmune disease that is a progressive, chronic, and inflammatory cause of cartilage and bone destruction due to a hyperplastic synovial membrane [1]. The prevalence of RA is approximately 5 per 1000 adults worldwide. This disease affects women 2 to 3 times more often than men and occurs at any age. RA leads to disability, the inability to work, increased mortality and socioeconomic costs [1,2]. The goal of treatment for RA patients is to relieve pain, control inflammation and achieve remission [3]. A variety of biologic agents targeting proinflammatory cytokines, such as tumor necrosis factor (TNF)-α, IL-1β, interferon (IFN)-γ and IL-6, have been suggested to be superior to conventional disease-modifying antirheumatic drugs (DMARDs). However, some RA patients are still refractory to biologic agents, as well as DMARDs. Therefore, new therapeutic strategies for RA need to be developed [4].

The fruit of *Psoralea corylifolia* L. (PCL) is widely used in traditional Chinese medicine as a kind of kidney-tonifying herb according to the traditional Chinese medicine theory. Recent research showed that the bavachin and bakuchiol of PCL have protective effect on bone loss in osteoporosis [5]. Previous studies have shown that PCL and its major constituents may have effects on the treatment of Alzheimer’s disease, Parkinson’s disease, eczema and psoriasis [6]. Recent research has shown that the coumarin in *P. corylifolia* L. could stimulate local new bone formation by enhancing the functions of osteoblasts [7]. However, research on the use of PCL in RA is lacking.

A variety of proinflammatory cytokines have been involved in the establishment of RA inflammation [8]. The imbalance of immunity between effectors and suppressors is one of the most essential factors in the development of RA. The main proinflammatory cytokines include TNF-α, IL-17A, IL-6, and IFN-γ produced by infiltrating T cells and other proinflammatory immune cells [4,8]. On the other hand, regulatory T cells and myeloid-derived suppressor cells (MDSCs) are the suppressive forces to inhibit inflammation. Reduction of regulatory T cells has been implied to be associated with the initiation and progression of RA [9,10]. MDSCs, as a heterogeneous population of early myeloid progenitors, immature granulocytes, macrophages and dendritic cells, are able to suppress immune responses [11,12]. These cells were first reported in a lung cancer model in 1987 [13], and MDSCs were thought of as bone marrow-derived cells that inhibited T cell proliferation. Over the past decades, researchers have mainly focused on the role of MDSCs in the field of cancer research [14]. The expansion of MDSCs has been reported to inhibit autoimmunity, which was associated with the amelioration of experimental autoimmune encephalomyelitis and reduced disease severity accompanied by significant inhibition of Th1 and Th17 immune responses and delayed disease onset through the inhibition of encephalitogenic Th1 and Th17 immune responses [15].

Collagen-induced arthritis (CIA) is an experimental autoimmune disease that is widely used as a model of RA [16]. In the present study, we used CIA as an animal model to mimic human RA. We aimed to investigate the effects and mechanisms of concentrated herbal granules of PCL on RA to provide some insights for future development of new drug. We found that PCL granules have the potential antiarthritic activity mediated by the anti-inflammatory effects. These findings indicate that PCL may be a promising therapeutic agent for the treatment of RA.

## 2. Materials and Methods

### 2.1. Preparation of PCL

**Figure 1:**
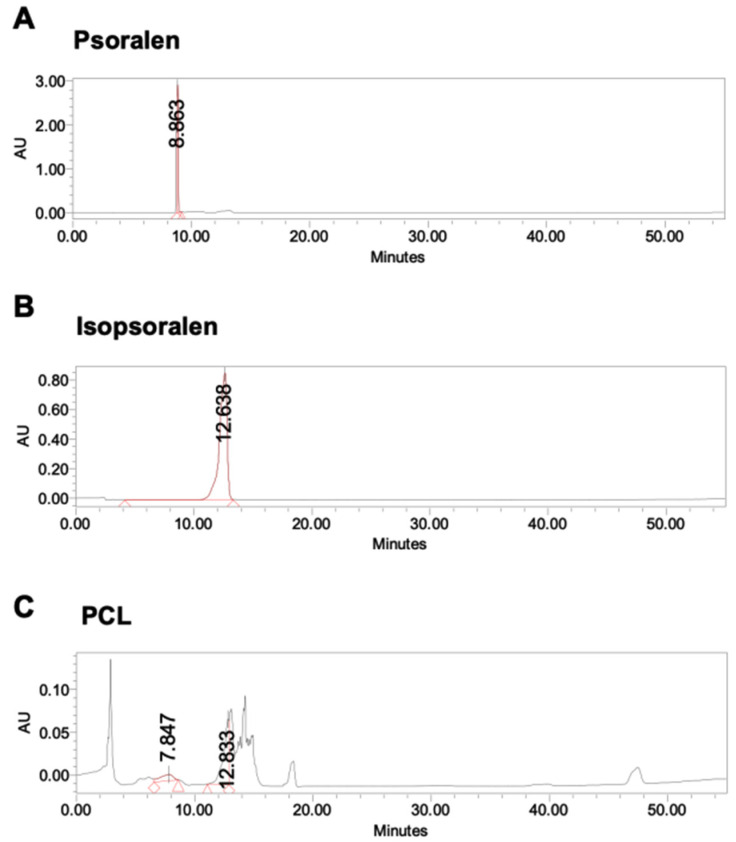
The HPLC profile of PCL. The chemical profile of PCL was determined using an RP-18 column and detected in the UV range at 280 nm. HPLC chromatograms of the reference compounds (**A**) psoralen, (**B**) isopsoralen and (**C**) PCL.

Herbal granules of PCL were purchased from Chuang Song Zong Pharmaceutical Co., Ltd. (Kaohsiung, Taiwan). The high-performance liquid chromatography (HPLC) profile of PCL is shown in Figure 1. A stock of this batch of herbal powder was kept in the herbarium at the School of Chinese Medicine, China Medical University, Taichung, Taiwan. The suggested dose of PCL in human is 1 gm per day. The dose of PCL in mouse was determined according to the Km factor. Therefore, the dose of PCL in mouse was as follows: body weight of mice: PCL weight = 20 gm:4 mg (according to the body weight of the mouse every other day). PCL was dissolved in 0.9% normal saline and orally administered at a dose of 20 μL/mouse/time/day.

### 2.2. Preparation of Starch (Starch from Corn)

Starch (corn) was purchased from Sigma-Aldrich (St. Louis, MO, USA) and used at as follows: body weight of mice: PCL weight = 20 gm:4 mg (according to the body weight of the mouse every other day). Starches were dissolved in 0.9% normal saline and orally administered at a dose of 20 μL/mouse/time/day.

### 2.3. Induction of CIA

Experimental CIA was induced as reported previously [16,17]. We used 8- to 12-week-old female DBA/1J mice (The Jackson Laboratory, USA) that were maintained in a standard animal facility and given food and water ad libitum. For each batch of experiments, 20 mice were sub-grouped into control group (*n* = 5), CIA group (*n* = 5), CIA + Starch group (*n* = 5) and CIA + PCL group (*n* = 5). We mixed Freund’s incomplete adjuvant (BD, Franklin Lakes, NJ, USA) with *M. tuberculosis* H37 Ra (BD, USA) to be Freund’s complete adjuvant (CFA) in a final concentration of 4 mg/mL. Bovine type II collagen was dissolved in 0.05 M acetic acid to a final concentration of 2 mg/mL. Subsequently, the bovine type II collagen acetic acid solution was emulsified with CFA at a ratio of 1:1 to 1 mg/mL. All CIA mice were intradermally immunized at the base of the tail with 100μL emulsion on day 0. On day 21 after the initial collagen immunization, the mice were intradermally boosted with 100 uL of bovine type II collagen emulsified in Freund’s incomplete adjuvant (IFA) [16,17]. All mice were treated in accordance with the Regulations and Guidelines on Scientific and Ethical Care and Use of Laboratory Animals of the Science Council. The study protocol was approved by the Animal Care and Use Committee of China Medical University, Taiwan (approval number: 103–172-N).

### 2.4. CIA Severity Score

During the course of CIA, arthritis severity was assessed every 2 days by clinical scoring ranging from 0 to 4 points for each paw. Each mouse could have a maximum of 16 points, which was determined as follows: 0 = no evidence of erythema or swelling; 1 = erythema and mild swelling confined to the mid-foot (tarsals) or ankle joint; 2 = erythema and mild swelling extending from the ankle to the mid-foot; 3 = erythema and moderate swelling extending from the ankle to the metatarsal joints; and 4 = erythema and severe swelling encompassing the ankle, foot, and digits, as described elsewhere [16].

### 2.5. Histological Assessment of Arthritis

At sacrifice, the isolated joints were dissected and fixed in buffered formaldehyde, decalcified in 10% ethylenediaminetetraacetic acid, and embedded in paraffin. Serial sections (3 μm) were stained with hematoxylin and eosin (H&E) to evaluate synovial inflammation. Sagittal sections were scored from 0 to 3 by a pathologist (Dr. John Wang, China Medical University Hospital, Taiwan) in a blinded manner by assessing inflammation and joint destruction. Histopathological changes in the joint were evaluated based on histologic parameters using a 0- to 3-point scale: 0 = clear joint cavity and no fusion; 1 = minimal synovitis without cartilage erosion; 2 = synovitis with focal proliferation and marginal erosion; and 3 = extensive synovial proliferation and adhesion to cartilage (Supplementary Figure S1). The sections were examined under a photomicroscope with an Aperio CS2 image capture device (Leica, Wetzlar, Germany).

### 2.6. Immunohistochemistry (IHC)

Joint tissues were fixed in 4% paraformaldehyde and then embedded in paraffin. Joint tissues were cut into 3 μm thick coronal sections. The sections were first incubated with primary antibodies against IL-17 (Santa Cruz Biotechnology, Dallas, TX, USA) overnight at 4 °C and then incubated with biotinylated secondary antibodies and avidin-biotinylated enzyme complex. DAB substrate was used as the detection reagent (Code K4065, CA, USA). The sections were examined under a photomicroscope with an Aperio CS2 image capture device (Leica, Germany). Histology scores were determined in a blinded manner by a pathologist (Dr. John Wang, China Medical University Hospital, Taiwan). Immunohistochemical changes in the joint were evaluated based on histologic parameters using a 0- to 3-point scale: 0 = <10% of the base of synovial membrane; 1 = 10–49% of the base of the synovial membrane; 2 = 50–74% of the base of the synovial membrane; 3 = 75–100% of the base of the synovial membrane (Supplementary Figure S2). The sections were examined under a photomicroscope with an Aperio CS2 image capture device (Leica, Germany).

### 2.7. Enzyme-Linked Immunosorbent Assay (ELISA)

TNF-α (BD, USA), IL-17A (BioLegend, San Diego, CA, USA), IL-6 (BD Biosciences, Franklin Lakes, NJ, USA), IFN-γ (BD Biosciences, Franklin Lakes, NJ, USA), IL-10 (BioLegend, San Diego, CA, USA), IgG1 (eBioscience, San Diego, CA, USA), IgG2a (BD Biosciences, Franklin Lakes, NJ, USA) and IgG2b (eBioscience, San Diego, CA, USA) levels in the culture supernatants were measured using sandwich ELISA and an ELISA microplate reader at 450 nm (BioTek, Winooski, VT, USA).

### 2.8. Flow Cytometry

Spleens and lymph nodes were isolated from mice in each group, and 1 × 10^6^ cells were incubated with PMA (50 ng/mL) plus ionomycin (500 ng/mL) for 5 h at 37 °C. The harvested cells were fixed and permeabilized (CytoFix/CytoPerm kit; BD Biosciences, Franklin Lakes, NJ, USA) and stained with fluorescence-labeled anti-CD4 (BD Biosciences, Franklin Lakes, NJ, USA), anti-IL-17A (BioLegend, San Diego, CA, USA), anti-TNF-α (BioLegend, San Diego, CA, USA), anti-IFN-γ (BD Biosciences, Franklin Lakes, NJ, USA), anti-Foxp3 (eBioscience, San Diego, CA, USA), anti-CD11b (BioLegend, San Diego, CA, USA), and anti-Gr-1 (BioLegend, San Diego, CA, USA) monoclonal antibodies. The expression of these markers was analyzed using a FACSVerse instrument and FlowJo 10.6.1 software (FlowJo LLC, Ashland, OR, USA).

### 2.9. Statistical Analysis

According to previous findings, DBA1/J strain is quite susceptible to CIA induction [16,17]. Previous studies also found that 5–6 mice per group would be enough to observe the effects of various herbal extracts or compounds in the CIA model [18,19]. To minimize the sacrifice of mice, we chose 5 mice per group for this study. The results are expressed as mean ± SEM (*n* = 5). Two-way ANOVA with the Bonferroni *post hoc* test of variance was used to indicate the statistical significance of the clinical scores, paw swelling and body weight among the means of the experimental groups. The results are shown as the mean ± SEM of the number of individual values. One-way Bonferroni *post hoc* analysis of variance was used to indicate the statistical significance of the results of H&E staining, IHC staining, ELISA and flow cytometric analysis of CD4 + IL17A+, CD4 + TNF-α+, and CD4 + IFN-γ+ cells among the means of the experimental groups. One-way analysis with Tukey *post hoc* test of variance was used to examine the flow cytometric data for MDSCs (CD11b + Gr1+). A *p*-value of less than 0.05 was considered statistically significant. Statistical analyses were carried out using GraphPad Prism version 6.0 (GraphPad Software, San Diego, CA, USA).

## 3. Results

### 3.1. The HPLC Profile of PCL

The HPLC profile of PCL. A hot water extract of PCL was prepared by dissolving in MeOH (2.0 mg/mL). Sample and standard solutions of psoralen and isopsoralen were filtered with a filter cartridge (pore size of 0.22 μm) prior to analysis. Chemical content quantification was performed on a Shimadzu HPLC system equipped with a Shimadzu LC-20AT pump, Shimadzu SIL-20 autosampler, and Shimadzu SPD-M20A detector. The HPLC profile of PCL was determined on an RP-18 column (Galaksil BF-C18B, 4.6 × 250 mm, 5 mm) at a flow rate of 1.0 mL/min with the detection of UV absorbance at 280 nm. The injection volume was 10 mL. The mobile phase was composed of a 0.1% TFA solution in water and methanol. The solvent gradient was as follows: 0–80 min from 10% methanol to 38% methanol. By diluting the stock solution, a series of standard solutions at the concentrations 0.5, 0.25, 0.125, 0.0625, and 0.0312 were prepared and used to calculate the concentrations of the examined compounds (Figure 1).

### 3.2. Treatment with PCL Ameliorates Clinical Severity in CIA Mice

**Figure 2:**
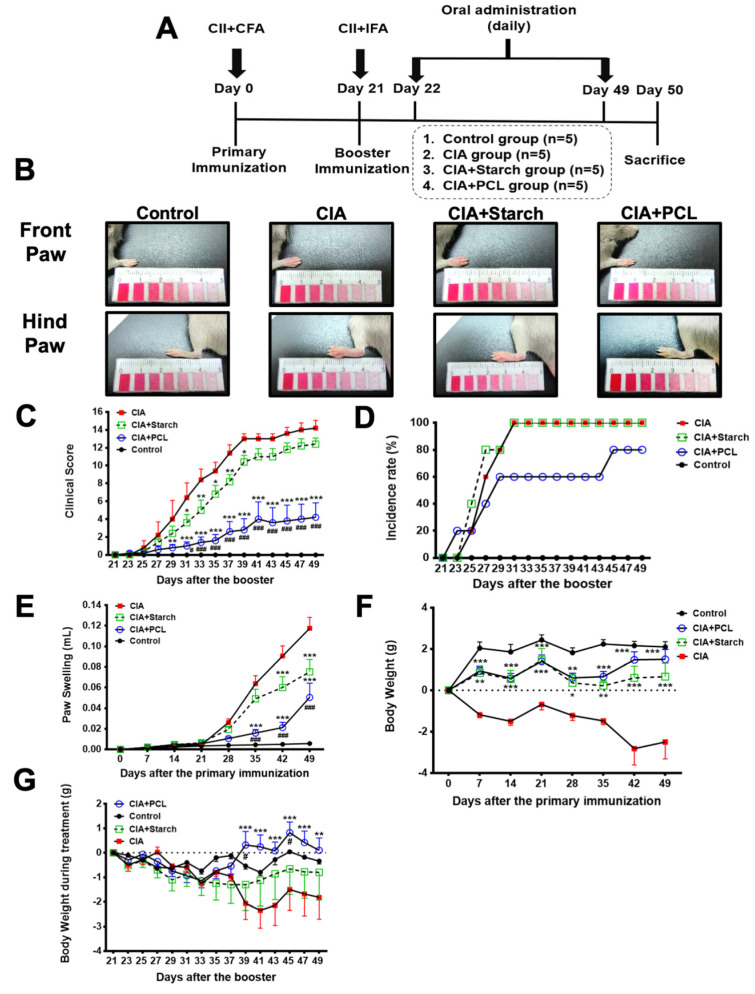
Treatment with PCL ameliorates clinical severity in CIA mice. DBA/1J mice were immunized with 100 µg of bovine type II collagen (CII) in Freund’s complete adjuvant. The mice were orally administered 200 mg/kg/day PCL or starch. (**A**) Chart showing the experimental design and treatment protocols. (**B**) Morphological changes in the diseased front and hind limbs. Front paws and hind paws are shown in the photos. (**C**) The severity of arthritis was evaluated by assigning a score of 0–4 per paw based on the degree of inflammation in each limb, with 0 indicating no arthritis and 4 indicating severe arthritis, for a maximum score of 16 per mouse. (**D**) Incidence of disease in the mice. (**E**) Average changes in paw volume. (**F**) Average changes in body weight from day 0–day 49. (**G**) Average changes in body weight from day 21–day 49 during the treatment. The data represent the mean ± SEM (*n* = 5 per group). * *p* < 0.05, ** *p* < 0.01, *** *p* < 0.001 vs. CIA group; # *p* < 0.05, ### *p* < 0.001 vs. CIA + Starch group.

Our experimental design is shown in Figure 2A. The results showed that CIA mice that received PCL treatment (CIA + PCL) had significantly reduced clinical scores, disease incidence and paw volumes compared with those in the CIA group and CIA + Starch treatment group (Figure 2B–E). The body weights of CIA mice treated with PCL were significantly increased compared with those of the CIA + Starch-treated group and the nontreatment group (Figure 2F). In addition, we also measured the body weight from day 21 to day 49 (Figure 2G). PCL did not cause weight loss. Consistent with the effective treatment of CIA, the clinical scores, incidence rate, and measurements of paw swelling indicated that PCL was highly effective in reducing joint inflammation. Detailed recordings of the front paws and hind paws in each group are shown in Supplementary Figure S3.

### 3.3. Treatment with PCL Reduces Joint Damage in CIA Mice

**Figure 3:**
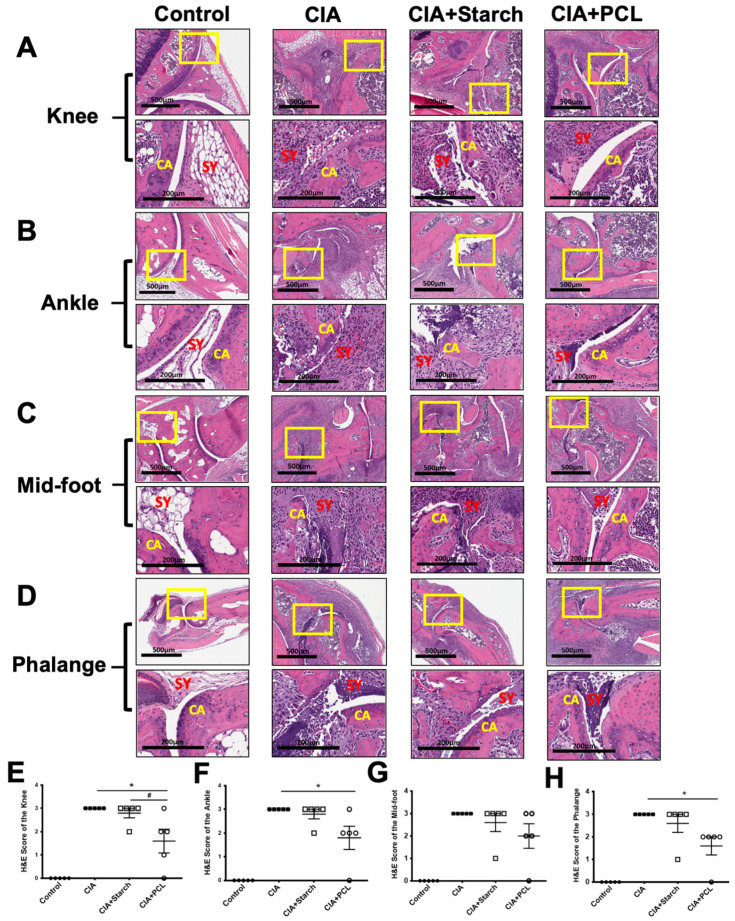
Histological parameters in the diseased hind limb joints. Hematoxylin and eosin staining (H&E staining) of representative mouse hind paws. CIA mice showed hyperplasia of the synovial tissue and increased synovitis and cartilage erosion. (**A**) Knee, (**B**) Ankle, (**C**) Mid-foot, and (**D**) Phalange in each group. The data are presented in (**E**–**H**) as the mean ± SEM (*n* = 5 per group), * *p* < 0.05 vs. CIA group; # *p* < 0.05 vs. CIA + Starch group. SY stands for the position of synovial membrane. CA represent for the position of cartilage.

Histological changes in CIA mouse joints were examined and are shown in Figure 3. H&E staining showed that the joints of CIA mice exhibited severe inflammatory cell infiltration in the hyperplastic synovium and cartilage and bone erosion compared with those of control mice. The joints of mice treated with 200 mg/kg/day PCL showed decreased inflammatory cell infiltration, slight synovial hyperplasia, cartilage destruction and bone erosion in the knee, ankle, mid-foot (tarsal and meta-tarsal) and phalange (Figure 3A–D). Statistically, the CIA + PCL group had reduced histological scores in the knee, ankle, mid-foot (tarsal and meta-tarsal) and phalange (Figure 3E–H).

**Figure 4:**
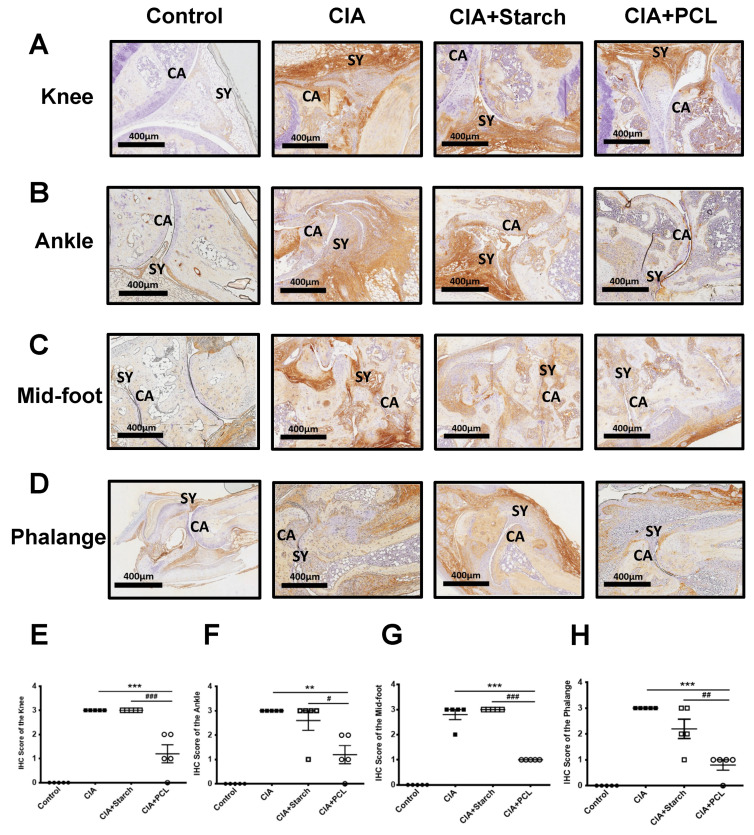
Histological parameters in the diseased hind limb joints. Immunohistochemical analysis of IL-17 in the joints of mice in each group. The levels of IL-17 in mouse joints after PCL treatment. IL17A is strongly positive for synovial cartilage markers (50–100%) in inflammatory areas. (**A**) Knee, (**B**) Ankle, (**C**) Mid-foot, and (**D**) Phalange in each group. The data are presented in (**E**–**H**) as the mean ± SEM (*n* = 5 per group), ** *p* < 0.01, *** *p* < 0.001 vs. CIA group; # *p* < 0.05, ## *p* < 0.01, ### *p* < 0.001 vs. CIA + Starch group. SY stands for the position of synovial membrane. CA represent for the position of cartilage.

The intensity of IL-17A-positive immunohistochemical staining in the CIA + PCL-treated group was significantly decreased compared with that in the CIA group (Figure 4A–D). Statistically, the CIA + PCL group exhibited a significant reduction in staining intensity compared with that in the CIA group or CIA + Starch group (Figure 4E–H).

### 3.4. Treatment with PCL Suppresses Proinflammatory Cytokines in CIA Mice

**Figure 5:**
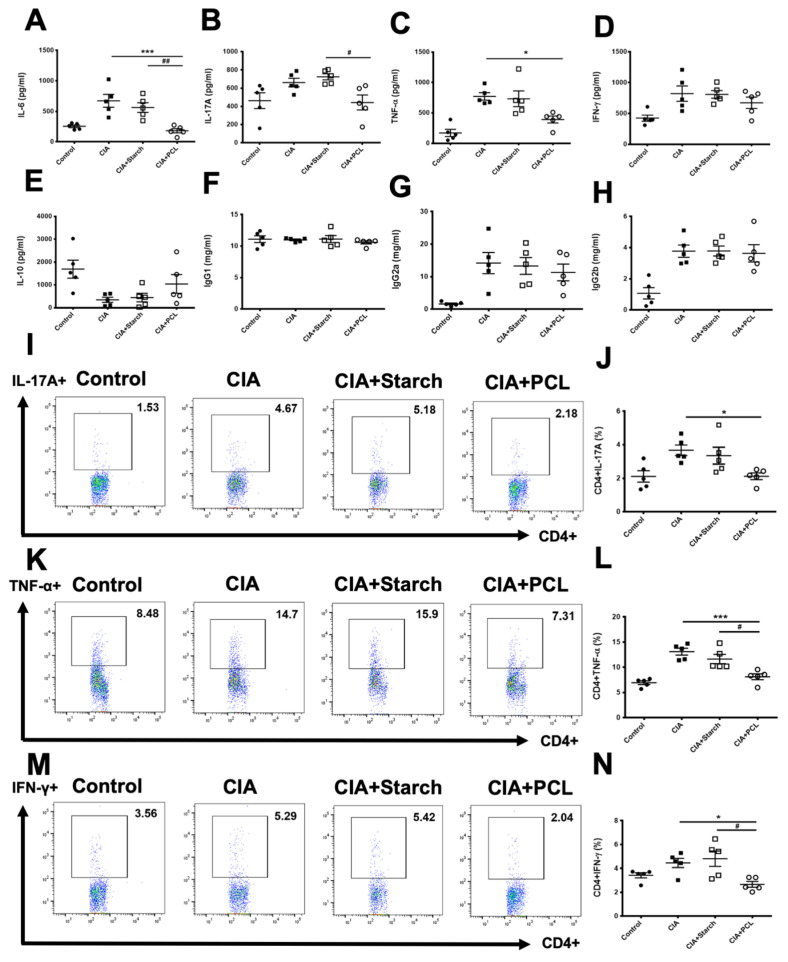
Changes in cytokines in the serum of CIA mice were measured by ELISA. (**A**) IL-6 (**B**) IL-17A (**C**) TNF-α (**D**) IFN-γ (**E**) IL-10 (**F**) IgG1 (**G**) IgG2a (**H**) IgG2b levels. Changes in cytokine production in splenocytes from CIA mice were measured by flow cytometry at day 50. (**I**,**J**) Changes in cytokines in CD4 + IL-17A+ splenocytes from CIA mice were measured by flow cytometry. (**K**,**L**) Changes in cytokines in CD4+ TNF-α+ of splenocytes from CIA mice were measured by flow cytometry. (**M**,**N**) Changes in cytokines in CD4 + IFN-γ+ splenocytes from CIA mice were measured by flow cytometry. The data are presented as the mean ± SEM (*n* = 5 per group), * *p* < 0.05, *** *p* < 0.001 vs. CIA group; # *p* < 0.05, ## *p* < 0.01, vs. CIA + Starch group.

We next measured serum levels of cytokines in CIA mice by ELISA (Figure 5A–H). The CIA + PCL group had significant reductions in the serum levels of IL-6 (Figure 5A) and IL-17A (Figure 5B) compared with the CIA + Starch group. The CIA + PCL group also had a significant reduction in the serum level of TNF-α (Figure 5C) compared with that in the CIA group. However, there was no significant difference in the level of IFN-γ (Figure 5D). The level of IL-10 seemed to be upregulated in the CIA + PCL group but the difference was not significant (Figure 5E). The level of IgG1, IgG2a and IgG2b were not different among the CIA, CIA + Starch and CIA + PCL groups (Figure 5F–H). We further used intracellular staining to investigate the levels of CD4 + IL-17A+, CD4 + TNF-α+ and CD4 + IFN-γ+ isolated from the spleen on day 50. The results showed that the CIA + PCL group had significant reductions in the percentages of CD4 + IL-17A+ cells compared to those in the CIA group (Figure 5I,J). The CIA + PCL group also had reductions in the percentages of CD4 + TNF-α+ (Figure 5K,L) and CD4 + IFN-γ+ (Figure 5M,N) cells compared to those in the CIA group or CIA + Starch group. However, the percentages of CD4 + Fop3+ cells were not significantly different among each group (data not shown). Taken together, these results suggested that PCL could reduce proinflammatory cytokines.

### 3.5. Treatment with PCL Upregulates MDSCs in CIA Mice

**Figure 6:**
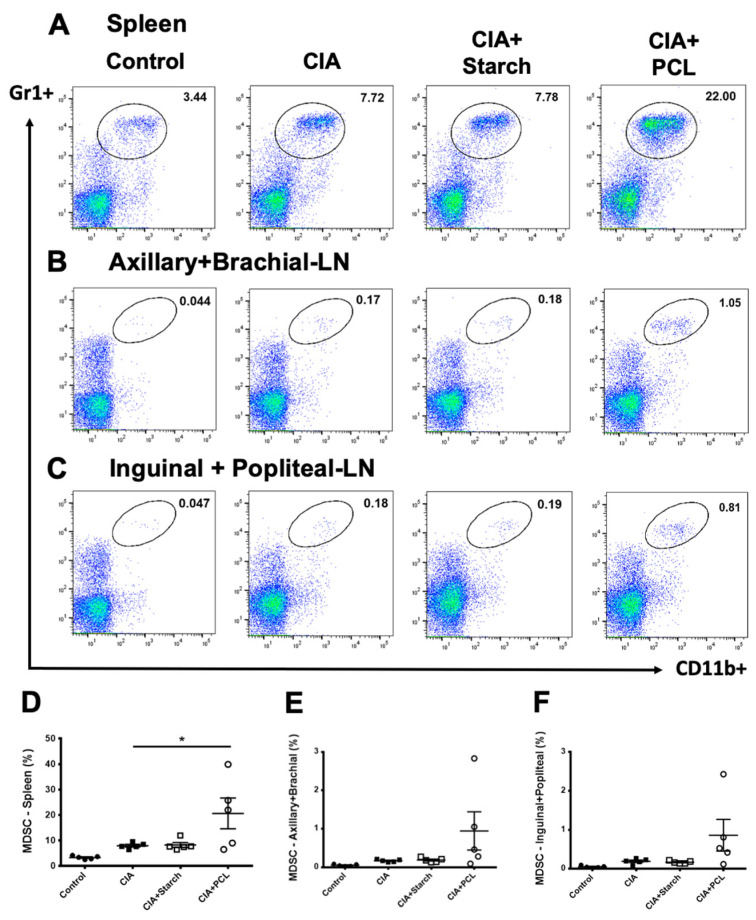
Changes in myeloid-derived suppressor cells in the spleen and lymph nodes of CIA mice. Immune cells isolated from the (**A**) spleen, (**B**) lymph nodes of axillary and brachial tissues, and (**C**) inguinal and popliteal lymph nodes were stained with anti-Gr1+ anti-CD11b+ and analyzed by flow cytometry. The percentages of MDSCs in the (**D**) spleen, (**E**) lymph nodes of axillary and brachial tissues, and (**F**) inguinal and popliteal lymph nodes in each group are presented as the mean ± SEM (*n* = 5 per group), * *p* < 0.05 vs. CIA group.

To analyze the level of MDSCs, we stained Gr1 and CD11b expression on the cellular surface as the surrogate marker of MDSCs. Cells were isolated from the spleen and lymph nodes of the axillary + brachial (representing draining lymph nodes of fore paws) and inguinal + popliteal (representing draining lymph nodes of hind paws) regions and analyzed by flow cytometry on day 50 in each group (Figure 6A–C). The results showed that the percentage of MDSCs was significantly higher in the PCL treatment group than in the CIA group (Figure 6D). There was an increasing trend in the percentage of MDSCs in draining lymph nodes, although the difference was not statistically significant (Figure 6E,F). We further calculated the absolute number of the MSCSs as shown in Table 1.

## 4. Discussion

In this study, we explored the role of PCL, a traditional Chinese herbal medicine, in the treatment of RA in a murine CIA model. Compared to starch, PCL significantly ameliorated the clinical symptoms and decreased paw swelling. Histopathology of the hind paws showed that PCL mitigated the infiltration of lymphocytes and destruction of synovial tissues. There were significant differences in the serum levels of TNF-α, IL-6 and IL-17A, as measured by ELISA, and the percentages of CD4 + IL-17A+, CD4 + TNF-α+, CD4 + IFN-γ+ T cells, as measured by flow cytometry. Finally, we also found that PCL treatment increased the percentage of MDSCs in the CIA disease model.

Early treatment can substantially slow the progression of joint damage in patients with RA, thereby preventing irreversible disability [1]. DMARDs, such as methotrexate and glucocorticoids, have been used for decades. New therapies, such as biologic agents, have been developed. However, the costs of biologic agents are considerable [20]. Furthermore, a significant proportion of RA patients still do not reach full remission, suggesting that new therapies are still needed [8,20]. In this study, we found that the concentrated PCL granules ameliorated clinical severities and reduced inflammation. It has been used in traditional Ayurvedic and Chinese medicine. Considering the lethal dose 50% (LD50) of psoralen, one of the ingredient compounds of PCL, is 625 mg/kg, the dosage of PCL used in this study is far less than the LD50 of psoralen [21]. However, further toxicity test is warranted before entering clinical studies. In addition, the PCL granules that we used in the present study is a kind of concentrated herbal granules containing starch as vehicle. Therefore, we use starch as a vehicle control in the experiments. It has to be mentioned that starch itself did not cause weight loss in the CIA model (Figure 2F,G). However, starch can rescue neither the pathological damage nor the inflammation. It is likely that although starch as a vehicle control might provide some carbohydrate nutrient to the CIA model, the major effect of the concentrated PCL granules is due to the ingredients of PCL instead of starch.

The major clinical characteristic of RA is joint swelling, which reflects inflammation in the synovial membrane [22]. The inflammatory cascade involves various immune cells, cytokines, chemokines and reactive oxidative stress and contributes to the critical immunopathologic damages in the joint environment [4]. During this process, the progressively activated effector T cells drive uncontrolled inflammation, leading to clinical symptoms of RA. These effector cells secret a variety of proinflammatory cytokines [4,8]. Multiple proinflammatory cytokines, such as TNF-α, IL-17, IFN-γ and IL-6, are critical factors that lead to the activation of endothelial cells and attract immune cells to accumulate within synovial tissues, resulting in joint destruction through cartilage erosion, bone damage and osteoclast activation [22]. These cytokines further attract other immune cells such as neutrophils to the local joints and further release high levels of oxidants and cytotoxic products to damage the synovial tissues and cartilages [4]. Our data showed that PCL-treated CIA mice had a significantly lower percentage of effector T cells and reduced production of proinflammatory cytokines, as compared with CIA group and CIA + Starch group. We further verified the expression of IL-17A in the synovial membrane was reduced with milder pathological changes in the joint tissue. This finding is compatible with previous findings that targeting IL-17A might be a potential therapeutic approach for chronic inflammation diseases or autoimmune disorders [23,24,25]. Further studies on the association of IL-17A and the joint damages are warranted. On the other hand, MDSCs are a heterogeneous population of early myeloid progenitors, immature granulocytes, macrophages and dendritic cells that are able to suppress immune responses [11]. In recent years, MDSCs have received considerable attention because they potently perturb both innate and adaptive immune responses [26]. MDSCs have the potential to suppress the autoimmune response, thereby limiting tissue injury [27]. MDSCs have now been shown to be an important population of immune suppressors in the pathogenesis of RA [27,28]. In this study, we demonstrated that PCL has dual functions and not only inhibits proinflammatory cytokines but also likely increases the number and percentage of MDSCs in the CIA model, which indicates that PCL is a potential immunomodulatory agent for the treatment of RA patients. However, it is necessary to indicate that there are a couple unsolved questions to be answered in the future. The exact role of PCL on MDSCs in CIA model need careful interpretation by other approaches such as functional studies. Since arginase-1 and inducible nitric oxide synthase are expressed by MDSCs [26], these are potential mechanisms that deserve future investigations.

Utilization of Chinese herbal medicine is not uncommon in Taiwan. Our previous study found that 27.3% of RA patients visited TCM clinics for consultations [29]. Among them, 76.4% took Chinese herbal medicines. Some of the patients also received acupuncture treatment for relief of discomforts [30]. These treatments are based on the accumulated experience of TCM doctors; future natural product-based drug discovery should also take Chinese herbal medicine into consideration [31]. It is also needed to provide more substantial evidence to demonstrate the efficacy and mechanism of TCM treatments.

Previous studies have shown that the main components of PCL include coumarins, flavonoids, and meroterpenes [6,32]. Metabolites of PCL that form after oral administration include psoralen, isopsoralen, psoralidin, bavachin, bavachinin, and others [6]. A previous study showed that the compound psoralen exhibited low cytotoxicity toward chondrocytes at a dose range of 1–10 μM. Psoralen suppressed the proliferation of chondrocytes at a dose of 100 μM [33]. However, the immunomodulatory effects of these compounds on RA have not yet been studied. The synergistic effect of different compounds may contribute to the antiarthritic effects of PCL that we investigated in the CIA mice, which warrants future investigations.

Our findings show that PCL is capable of significantly suppressing the levels of the proinflammatory cytokines IL-6, IL-17A and TNF-α and substantially mitigating paw swelling, with histopathologic evidence of decreased cartilage erosion and the amelioration of synovial tissue destruction. Perhaps most importantly, PCL might also have the potential to increase the number and percentage of MDSCs, which are upregulated in inflammatory arthritis and are therefore a critical aspect for consideration in the treatment of RA. Future investigations on these compounds to understand their effects and mechanism for the treatment of RA are warranted.

## 5. Conclusions

In summary, our study data indicate that PCL, a traditional Chinese medicine, has therapeutic antiarthritic activity, according to our testing of a murine CIA model. PCL is capable of suppressing the levels of the proinflammatory cytokines and substantially mitigating clinical severity. PCL could be a potential candidate herb for the future development of RA therapy.

## Figures and Tables

**Table 1:** Average absolute number of MDSCs among each group. Data were shown in mean ± SEM (*n* = 5 per group) (Unit: cells per mouse).

		Spleen (*n* = 5)	Axillary + Brachial Lymph Nodes (*n* = 5)	Inguinal + Popliteal Lymph Nodes (*n* = 5)
Control	Average	899.42	13.80	13.83
	SEM	80.01	3.12	2.94
CIA	Average	2107.27	46.79	54.00
	SEM	123.11	4.08	8.77
CIA + Starch	Average	2198.67	51.43	47.11
	SEM	245.24	7.30	4.50
CIA + PCL	Average	5599.02	261.00	239.58
	SEM	1675.46	137.26	116.68

## Data Availability

The data that support the findings of this study are available from the corresponding author upon reasonable request.

## Supplementary Materials

The following are available online at https://www.mdpi.com/article/10.3390/life11060587/s1, Figure S1: Histological assessment of arthritis, Figure S2: Immunohistochemistry of arthritis, Figure S3: Clinical phenotypes of the front paws and hind paws in each group.
