# Supplementary material for: Psoralea corylifolia L. Ameliorates Collagen-Induced Arthritis by Reducing Proinflammatory Cytokines and Upregulating Myeloid-Derived Suppressor Cells
Source: Life (Basel). 2021 Jun 21;11(6):587. doi: 10.3390/life11060587 (PMC8235662; doi:10.3390/life11060587)
Supplement: Supplementary file 1 [file life-11-00587-s001.zip › life-1206597-supplementary.pdf]

life

MDPI

Article

Psoralea corylifolia L. Ameliorates Collagen-Induced Arthritis by Reducing Proinflammatory Cytokines and Upregulating Myeloid-Derived Suppressor Cells

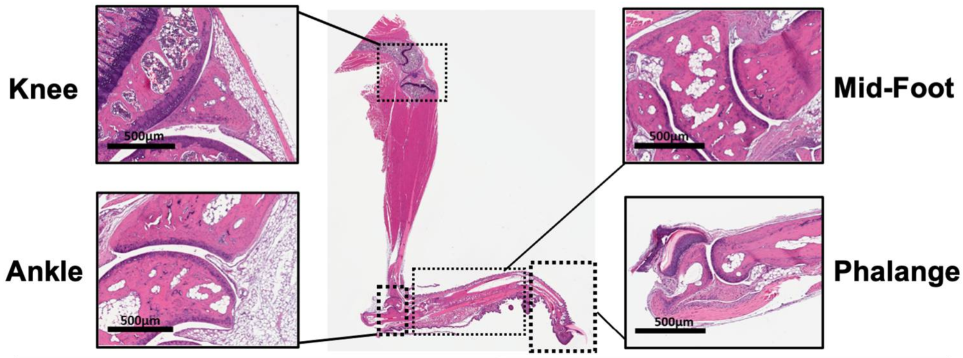

Score of the H & E Stain	For example
0 = clear joint cavity and no fusion	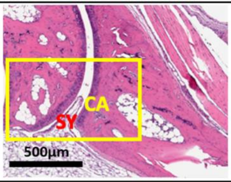
1 = minimal synovitis without cartilage erosion	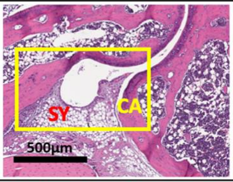
2 = synovitis with focal proliferation and marginal erosion	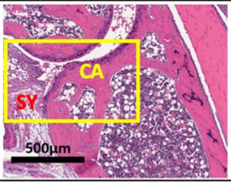
3 = extensive synovial proliferation and adhesion to cartilage	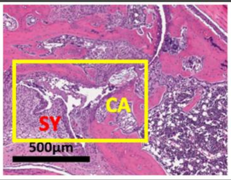

Figure S1. Histological assessment of arthritis. Hematoxylin and eosin staining (H&E staining) of representative hind paws, including knee, ankle, midfoot and phalange. We took ankle arthritis as an example to present the illustration. The pathologist used a 0- to 3- point scale to assess the histopathological changes of the joints according to histological parameters. 0 = clear joint cavity without fusion; 1 = mild synovitis without cartilage erosion; 2 = synovitis with local hyperplasia and edge erosion; 3 = extensive synovial hyperplasia and adhesion to cartilage. SY stands for the position of synovial membrane. CA represents for the position of cartilage.

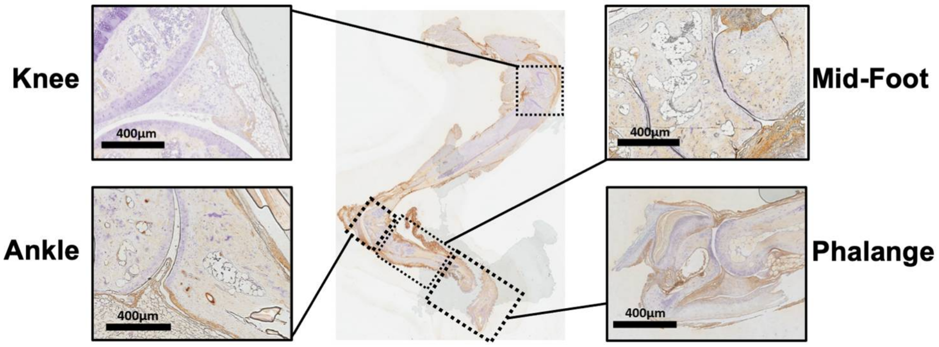

Score of the IHC Stain	For example
0 = <10% of the base of synovial membrane	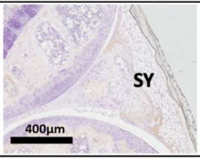
1 = 10-49% of the base of synovial membrane	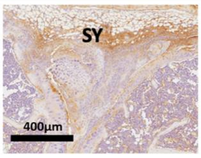
2 = 50-74% of the base of synovial membrane	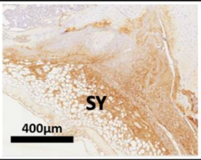
3 = 75-100% of the base of synovial membrane	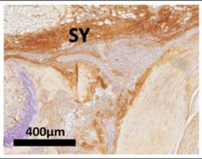

Figure S2. Immunohistochemistry of arthritis. Immunohistochemical analysis of IL-17A in the joints of representative hind paws, including knee, ankle, midfoot and phalange. We took knee arthritis as an example to present the illustration. The pathologist used a 0-to 3-point scale to assess the histopathological changes of the joints according to parameters of immunohistochemical analysis. 0 = <10% of the base of synovial membrane; 1 = 10 to 49% of the base of synovial membrane; 2 = 50 to 74% of the base of synovial membrane; 3 = 75 to 100% of the synovial membrane. SY stands for the position of synovial membrane.

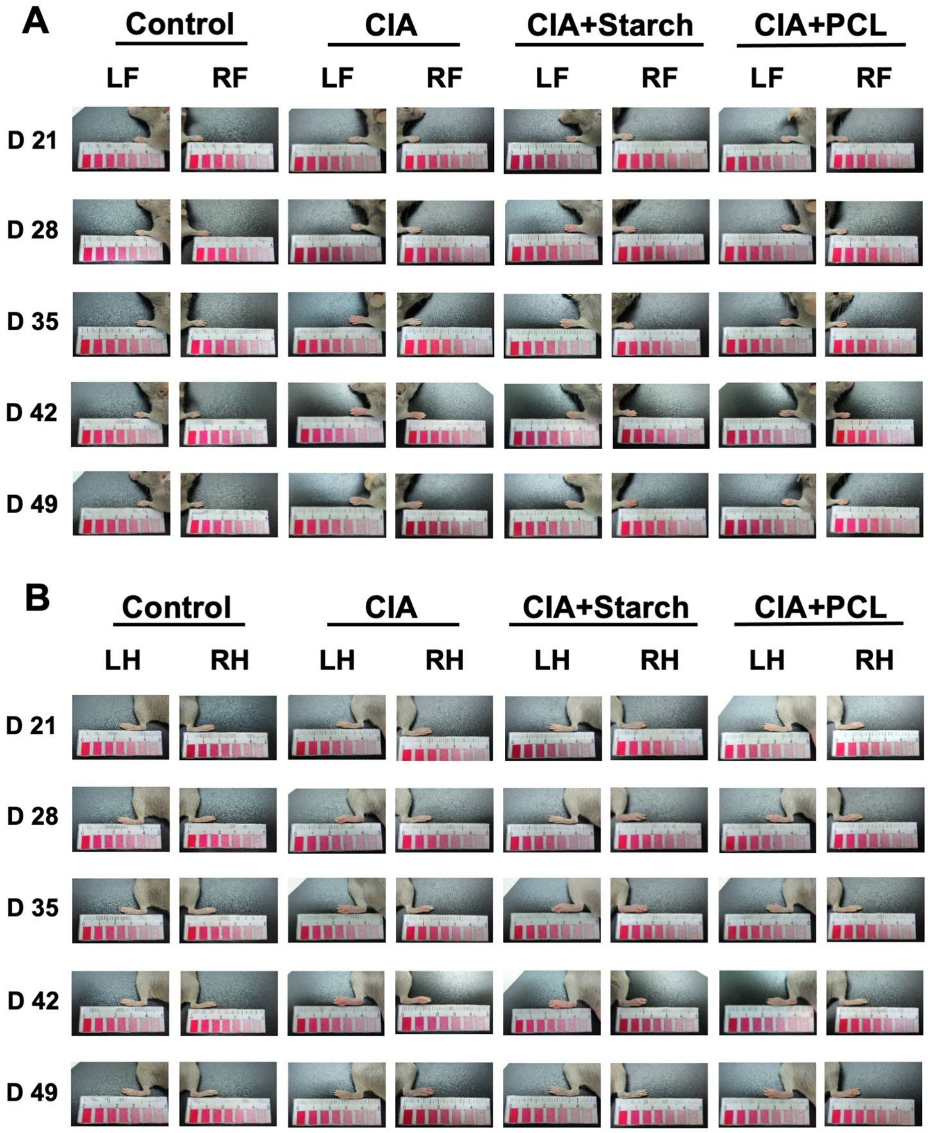

Figure S3. Clinical phenotypes of the front paws and hind paws in each group. Our experimental found that after booster immunization on D21, the limbs of CIA mice were significantly swollen. Therefore, we present the limb changes in each group of front paws and hind paws every 7 days from D21 to D49.
